# Medulloblastoma-associated DDX3 variant selectively alters the translational response to stress

**DOI:** 10.18632/oncotarget.8612

**Published:** 2016-04-05

**Authors:** Sekyung Oh, Ryan A. Flynn, Stephen N. Floor, James Purzner, Lance Martin, Brian T. Do, Simone Schubert, Dedeepya Vaka, Sorana Morrissy, Yisu Li, Marcel Kool, Volker Hovestadt, David T.W. Jones, Paul A. Northcott, Thomas Risch, Hans-Jörg Warnatz, Marie-Laure Yaspo, Christopher M. Adams, Ryan D. Leib, Marcus Breese, Marco A. Marra, David Malkin, Peter Lichter, Jennifer A. Doudna, Stefan M. Pfister, Michael D. Taylor, Howard Y. Chang, Yoon-Jae Cho

**Affiliations:** ^1^ Department of Neurology and Neurological Sciences, Stanford University School of Medicine, Stanford, CA, USA; ^2^ Department of Neurosurgery, Stanford University School of Medicine, Stanford, CA, USA; ^3^ Program in Epithelial Biology, Stanford University School of Medicine, Stanford, CA, USA; ^4^ Department of Molecular and Cell Biology, University of California, Berkeley, CA, USA; ^5^ Department of Developmental Biology, Stanford University School of Medicine, Stanford, CA, USA; ^6^ Department of Surgery, Division of Neurosurgery, University of Toronto, ON, Canada; ^7^ Developmental and Stem Cell Biology Program, The Hospital for Sick Children, Toronto, ON, Canada; ^8^ Department of Surgery, Division of Neurosurgery and Labatt Brain Tumour Research Centre, The Hospital for Sick Children, Toronto, ON, Canada; ^9^ Canada's Michael Smith Genome Sciences Centre, BC Cancer Agency, Vancouver, BC Canada; ^10^ Division of Pediatric Neurooncology, German Cancer Research Center (DKFZ), Heidelberg, Germany; ^11^ Division of Molecular Genetics, German Cancer Research Center (DKFZ), Heidelberg, Germany; ^12^ Department of Vertebrate Genomics, Max Planck Institute for Molecular Genetics, Berlin, Germany; ^13^ The Vincent Coates Foundation Mass Spectrometry Laboratory, Stanford University, Stanford, CA, USA; ^14^ Cancer Biology Program, Stanford University School of Medicine, Stanford, CA, USA; ^15^ Cancer Genetic Program, The Hospital for Sick Children, Toronto, ON, Canada; ^16^ Department of Chemistry, University of California, Berkeley, CA, USA; ^17^ Physical Biosciences Division, Lawrence Berkeley National Laboratory, Berkeley, CA, USA; ^18^ Howard Hughes Medical Institute, University of California, Berkeley, CA, USA; ^19^ Department of Laboratory Medicine and Pathobiology, University of Toronto, ON, Canada; ^20^ Howard Hughes Medical Institute, Stanford University School of Medicine, Stanford, CA, USA; ^21^ Papé Family Pediatric Research Institute, Department of Pediatrics, Oregon Health and Science University, Portland, OR, USA; ^22^ Knight Cancer Institute, Oregon Health and Science University, Portland, OR, USA

**Keywords:** medulloblastoma, DDX3X, DDX3, RNA helicase, CLIP-seq

## Abstract

*DDX3X* encodes a DEAD-box family RNA helicase (DDX3) commonly mutated in medulloblastoma, a highly aggressive cerebellar tumor affecting both children and adults. Despite being implicated in several facets of RNA metabolism, the nature and scope of DDX3′s interactions with RNA remain unclear. Here, we show DDX3 collaborates extensively with the translation initiation machinery through direct binding to 5′UTRs of nearly all coding RNAs, specific sites on the 18S rRNA, and multiple components of the translation initiation complex. Impairment of translation initiation is also evident in primary medulloblastomas harboring mutations in *DDX3X*, further highlighting DDX3′s role in this process. Arsenite-induced stress shifts DDX3 binding from the 5′UTR into the coding region of mRNAs concomitant with a general reduction of translation, and both the shift of DDX3 on mRNA and decreased translation are blunted by expression of a catalytically-impaired, medulloblastoma-associated DDX3^R534H^ variant. Furthermore, despite the global repression of translation induced by arsenite, translation is preserved on select genes involved in chromatin organization in DDX3^R534H^-expressing cells. Thus, DDX3 interacts extensively with RNA and ribosomal machinery to help remodel the translation landscape in response to stress, while cancer-related DDX3 variants adapt this response to selectively preserve translation.

## INTRODUCTION

The DEAD-box RNA helicases are a large family of multifaceted proteins that bind and remodel RNA and RNA–protein complexes in an ATP-dependent manner. In doing so, they facilitate multiple aspects of RNA metabolism ranging from transcription, splicing and transport to ribosome biogenesis, translation, and degradation [1]. *DDX3X*, which encodes a member of this family (DDX3), is especially intriguing given it is mutated recurrently in several cancers, most notably medulloblastoma (MB), a highly malignant cerebellar tumor. Indeed, genome sequencing studies by our group and others have identified *DDX3X* mutations in over 50% of Wingless (WNT) subgroup medulloblastomas in children and upwards of 60% of Sonic hedgehog (SHH) subgroup medulloblastomas in adults [2, 3]. Medulloblastoma-associated *DDX3X* mutations occur exclusively the helicase domains [3, 4] and have been shown (or are predicted) to result in catalytically impaired proteins with limited ATPase and helicase activity [4–6]. And though these mutants potentiate Wnt pathway activity when co-expressed with stabilized β-catenin [4], how the impairment of DDX3′s catalytic activity contributes to oncogenesis remains unclear given we know very little about the extent and nature of DDX3′s binding to RNA normally.

Previous studies have suggested DDX3 facilitates resolution of structurally complex 5′ untranslated regions (UTR) to facilitate translation [7]. Biochemical studies have further revealed that DDX3 and its homologs interact directly with the translation initiation machinery through eukaryotic initiation factors eIF4E and eIF4G [1] to influence translation globally [8]. However, the effect of DDX3′s interactions with the initiation machinery is debated as DDX3 has been shown to both enhance and inhibit translation [9–11]. DDX3 also contributes to formation of cytoplasmic stress granules (SG) [1], which sequester mRNAs in response to exogenous or endogenous stress and, with the exception of certain stress response genes, halt their translation [12, 13]. Indeed, SGs are characterized by an accumulation of stalled translation initiation complexes and likely triage coding RNAs under stress conditions while their fates are determined [12, 13]. Notably, knockdown of DDX3 impairs proper SG formation and overexpression of the DDX3 N-terminal eIF4F-binding domain is sufficient to induce SGs [9, 14–16]. Whether cancer-related mutations in *DDX3X* influence SG formation, and additionally, differentially target mRNAs under stress remains unknown [1].

## RESULTS AND DISCUSSION

### Transcriptome-wide binding analysis of DDX3

**Figure 1:**
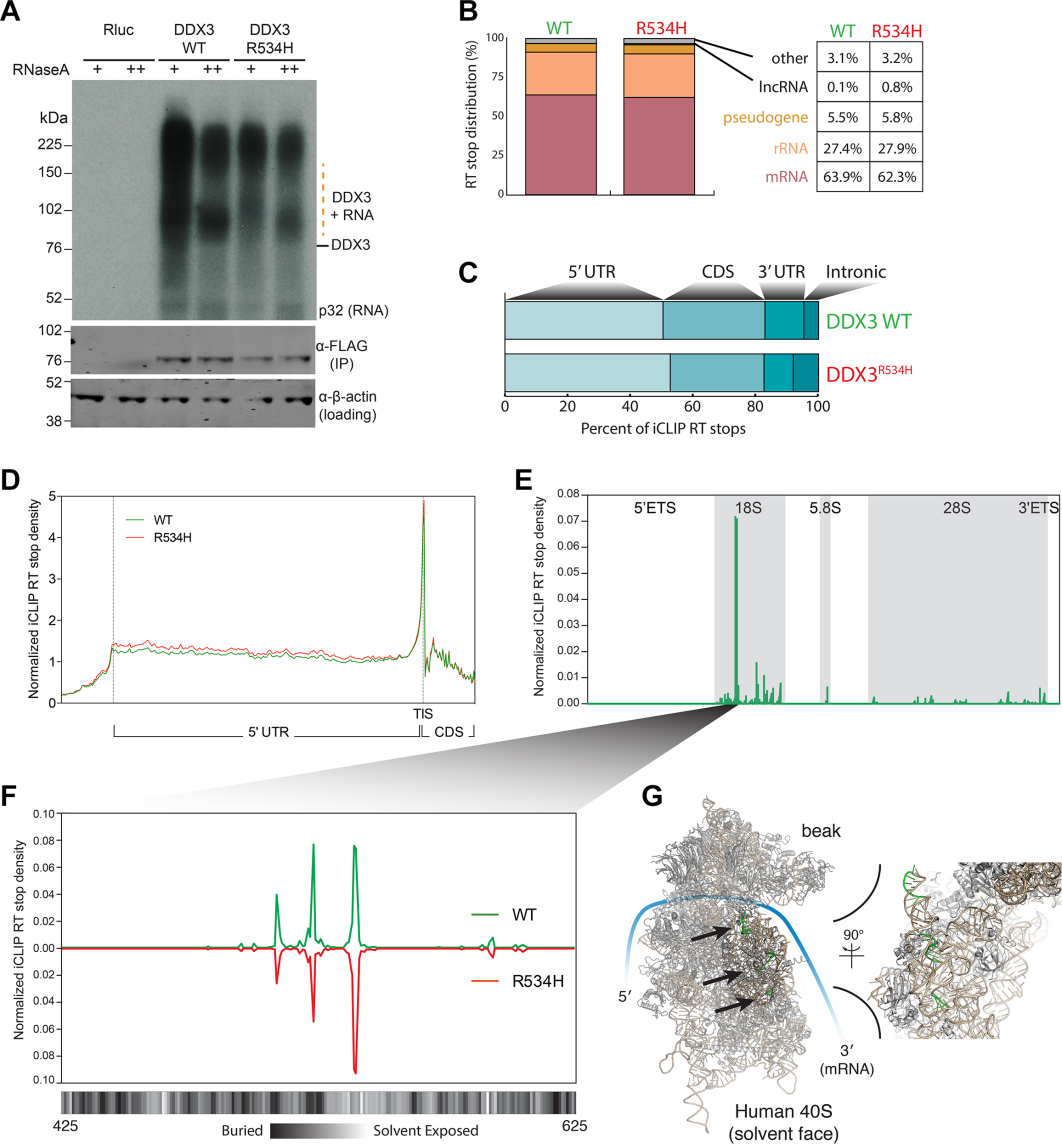
DDX3 binds the 5′ leader of mRNA and specifically interacts with the 18S rRNA (**A**) Autoradiogram of ^32^P labeled RNA UV-crosslinked to FLAG-DDX3 (top) and Western blot (WB) analysis of DDX3 enrichment (bottom). β-Actin, loading control. (**B**) Distribution of RT stops identified in the DDX3 and DDX3^R534H^ iCLIP experiments. Percentage of unique RNA crosslinked in each RNA class is noted. (**C**) Distribution of RT stops mapped to indicated mRNA regions. (**D**) Metagene analysis of normalized iCLIP RT stop density from DDX3 (green) or DDX3^R534H^ (red) experiments. Scaled 5′UTR regions and 50 nts up and downstream of the 5′UTR are plotted. TIS, translation initiation site. CDS, coding sequence. (**E**) Normalized iCLIP RT stop density from DDX3 and DDX3^R534H^ iCLIP mapping to the full rRNA transcribed region. (**F**) Zoom of e at the most well bound site on the 18S rRNA with the solvent accessibility of the 18S rRNA annotated below at single nucleotide resolution (calculated using PyMOL). (D–E) iCLIP RT stops were normalized for library size and calculated as counts in 10 million. (**G**) Structure of the human 40S ribosome with the three binding sites of DDX3 in f highlighted in green and indicated with arrows.

To identify the RNA targets of wild-type DDX3 and the impact of a catalytically defective DDX3 variant harboring a recurrent substitution at arginine 534 with histidine (DDX3^R534H^) [5] on RNA binding, we performed individual-nucleotide crosslinking and immunoprecipitation (FAST-iCLIP) [17] in HEK293 cell line, where a large volume of data for RNA-protein global interaction and translation landscape are available for direct comparison with our study. We expressed FLAG epitope-tagged DDX3 constructs expressed at near-endogenous levels (Supplementary Figure S1A) for 8 hours throughout this study to avoid overwhelming effect of overproduced exogenous DDX3 proteins. Tandem immunoprecipitations using anti-FLAG and anti-DDX3 antibodies of UV-crosslinked cell lysates and subsequent gel analysis revealed specific DDX3 RNA-protein complexes sensitive to RNase A (Figure 1A) and DDX3-bound RNAs were sequenced, and high confidence crosslinking sites (RT stops) identified [17]. Stringently filtered RT stops revealed a strong bias towards protein-coding mRNA binding (63.9% of mapped reads) and rRNA (27.4%), with pseudogene (5.5%), lncRNAs (0.1%), and other RNAs (3.1%) constituting the remainder (Figure 1B).

Although, DDX3^R534H^ reproducibly recovered less RNA per protein than WT (Figure 1A) and produced fewer RT stops per bound mRNA than DDX3 after normalizing for sequencing depth (Supplementary Figure S1B and Supplementary Table S1), the general binding profile and transcriptome targets were similar between DDX3 and DDX3^R534H^. Notably, we found DDX3 binds to a large fraction of the coding transcriptome (> 11,000 individual genes; Supplementary Table S1) and the number of iCLIP RT-stops per gene largely correlated with gene expression (Supplementary Figure S1C). Metagene analysis revealed binding across the 5′UTR (Figure 1C) with a robust enrichment of DDX3 directly at the translation initiation codon (TIS) (Figure 1D). We also noted DDX3 binding near transcription start sites (TSSs) of its target mRNAs (Supplementary Figure S1D), suggesting DDX3 gets loaded at the 5′ cap of its targets, consistent with its known interaction with mRNA-cap binding factors eIF4E and eIF4G [9, 14, 15]. Surprisingly, we also found DDX3 binds all three mature rRNAs (18S, 5.8S, and 28S) with particular enrichment on the 18S rRNA at three specific regions (Figure 1E–1G) [17]. These DDX3-bound regions involve both solvent exposed and buried nucleotides in the fully assembled ribosome [18] (Figure 1F–1G), suggesting DDX3 either interacts with the 18S rRNA along the maturation process or that these regions are flexible in the assembled 40S ribosome.

Although we did not identify a specific consensus DDX3 binding motif, we found DDX3 binds to sites high in GC content (Supplementary Figure S1E), supporting its proposed role in unwinding of secondary structures in mRNA 5′UTRs, and sequences 3′ to DDX3 binding sites were particularly high in guanidine (G) content (Supplementary Figure S1E). Notably, both DDX3 and DDX21 [19] iCLIP RT stops were positioned in overall G-rich sequences (Supplementary Figure S1E), suggesting that binding to regions of high guanidine content may be shared by DEAD-box proteins. In contrast, PCBP2 (Poly C Binding Protein 2) iCLIP RT stops [17] were found in the middle of cytosine (C)-rich sequences (Supplementary Figure S1E), reflecting its known target selection, and also uridine (U)-rich sequences (Supplementary Figure S1E). Thus, our experiments reveal that DDX3 directly contacts major RNA components of the translation initiation machinery with a predilection for guanidine-rich regions, and while the MB-related DDX3^R534H^ binds less avidly to RNA than wild-type DDX3, its cellular RNA target selection is largely preserved.

### Impaired translation is evident in *DDX3X*-mutated medulloblastoma gene expression signatures

**Figure 2:**
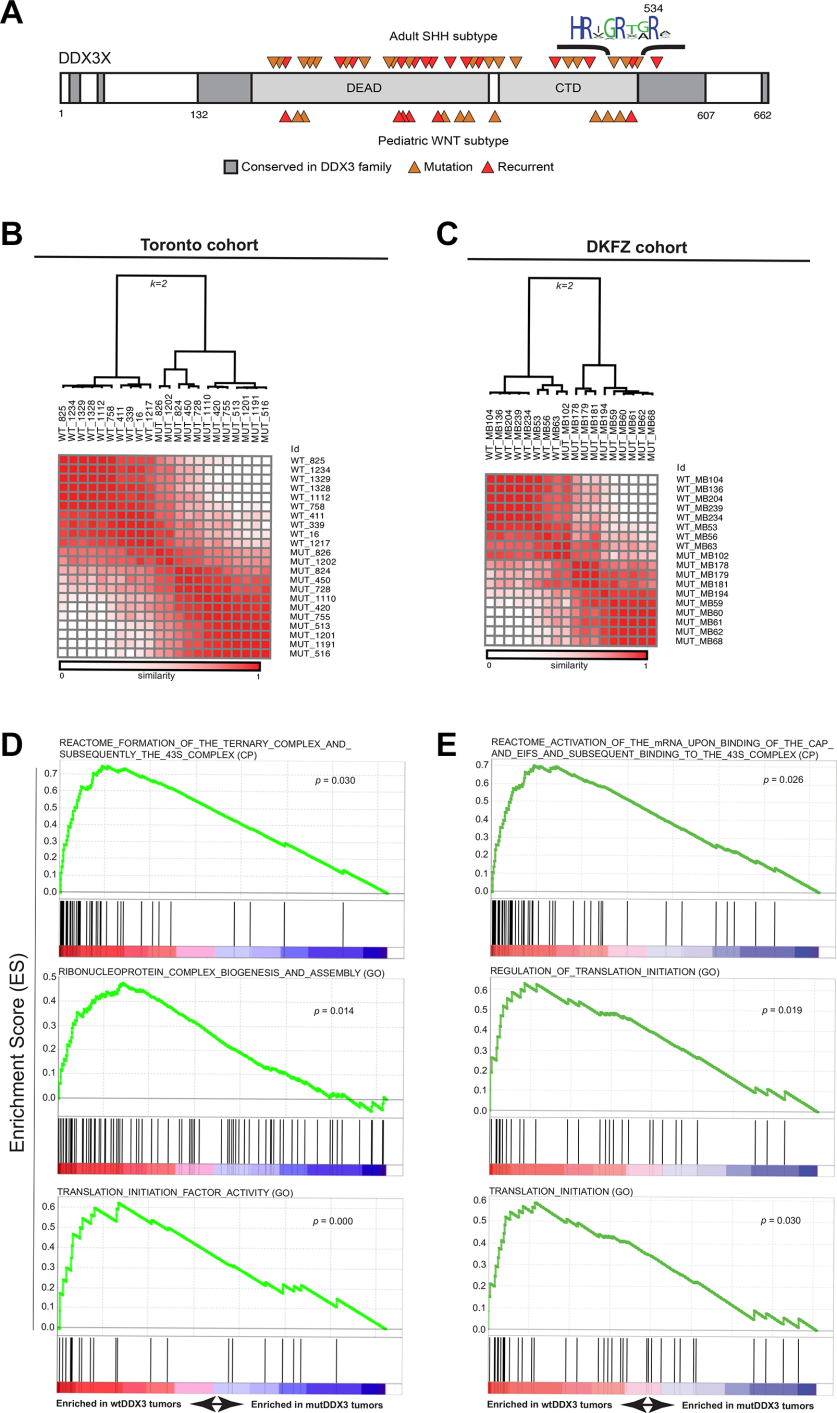
*DDX3X* mutations in SHH subtype MB exhibit impaired translation (**A**) Conserved regions in the DDX3 family are shown in dark gray with canonical DEAD-box protein domains in light gray. Medulloblastoma-associated mutations in either the SHH (top) or WNT (bottom) subtype are indicated by orange arrows with recurrent mutations colored red. (**B** and **C**) Unsupervised consensus clustering (*k* = 2) of RNA-seq-derived gene expression data (FPKM) from *DDX3X*-mutated and *DDX3X* wild type (WT) SHH-subtype medulloblastoma cases accrued from Toronto (A) or the German Cancer Research Center (DKFZ) (C). (**D** and **E**) Selected enrichment plots identifying genes associated with translation initiation and ribosome assembly enriched in wild-type relative to *DDX3X*-mutated medulloblastoma cases in both Toronto (C) and DKFZ (D) cohorts. GO x = gene ontology (MsigDB c5), CP = canonical pathways (MSigDB c2).

Without exception, *DDX3X* mutations reported in MB map to its helicase domains (Figure 2A), and are predicted to produce proteins with either amino acid substitution or in-frame deletion but not premature termination codons or frameshifts [3, 4], suggesting full-length, mutant protein is selected by MB tumors. Recent biochemical studies have demonstrated that most of these missense DDX3 variants found in MB, including DDX3^R534H^, have impaired catalytic activity [5, 6]. To examine whether MBs harboring mutations in *DDX3X* could be distinguished at the transcriptional level from those not carrying mutations, we analyzed whole transcriptome RNA sequencing (RNA-seq) of two non-overlapping cohorts of SHH-subtype MB patients (Tronto and DKFZ; Supplementary Table S2). Unsupervised consensus clustering showed *DDX3X*- mutated MBs clustered separate from their wild type (WT) counterparts in both cohorts (Figure 2B–2C, Supplementary Figure S2, and Supplementary Table S3). We next investigated the transcriptional profiles of WT- and mutant-*DDX3X* MBs for enrichment of specific biological pathways. Gene set enrichment analysis (GSEA) [20], performed independently on each dataset, revealed notable enrichment of genes involved in translation initiation in MBs with WT *DDX3X* relative to those harboring *DDX3X* mutations (Figure 2D–2E and Supplementary Table S3), suggesting mutations in *DDX3X* result in deregulated translation in MB.

Importantly, the gene expression signatures that reflect faulty translation initiation in *DDX3X*-mutated MBs were not due to altered association of catalytically-impaired DDX3 with components of the translation initiation complex, as immunoprecipitation (IP) of FLAG-tagged DDX3 (FLAG-DDX3) or DDX3^R534H^ (FLAG-DDX3^R534H^) followed by subsequent LC-MS/MS analysis revealed proteins co-purifying with FLAG-DDX3X^R534H^ largely overlapped with those recovered with FLAG-DDX3 (FDR < 0.01) (Supplementary Figure S3A–S3B). These proteins included a large fraction of eIF3 and eIF4 complex components, other DEAD-box proteins such as DDX5, DDX17, and DDX21, and other factors involved in translation and splicing (Supplementary Figure S3B and Supplementary Table S3). Thus, primary MBs with mutated DDX3 harbor gene expression changes reflecting impaired translation initiation, yet MB-associated DDX3 variants maintain extensive protein-protein interactions with components of the translation machinery.

### DDX3 catalytic activity is required for proper translation response to stress

Given the known role for DDX3 in regulating formation of stress granules (SG), we examined the effect of exogenous stress on DDX3-mRNA interactions [9, 14–16, 21]. SGs sequester mRNAs in response to exogenous or endogenous stress and, with the exception of certain stress response genes, halt their translation [12, 13]. Notably, knockdown of DDX3 has previously been shown to impair proper SG formation and overexpression of the DDX3 N-terminal eIF4F-binding domain is sufficient to induce SGs [9, 14–16]. In cells expressing DDX3^R534H^, we observed a mild increase in DDX3-positive granular structures in the absence of stress (Supplementary Figure S4C), though both DDX3 and DDX3^R534H^ robustly localized to large SGs in the presence of sodium arsenite (0.5 mM, 45 minute treatment) as measured by punctate cytosolic TIA-1 immunostaining (Supplementary Figure S4B–S4D).

**Figure 3:**
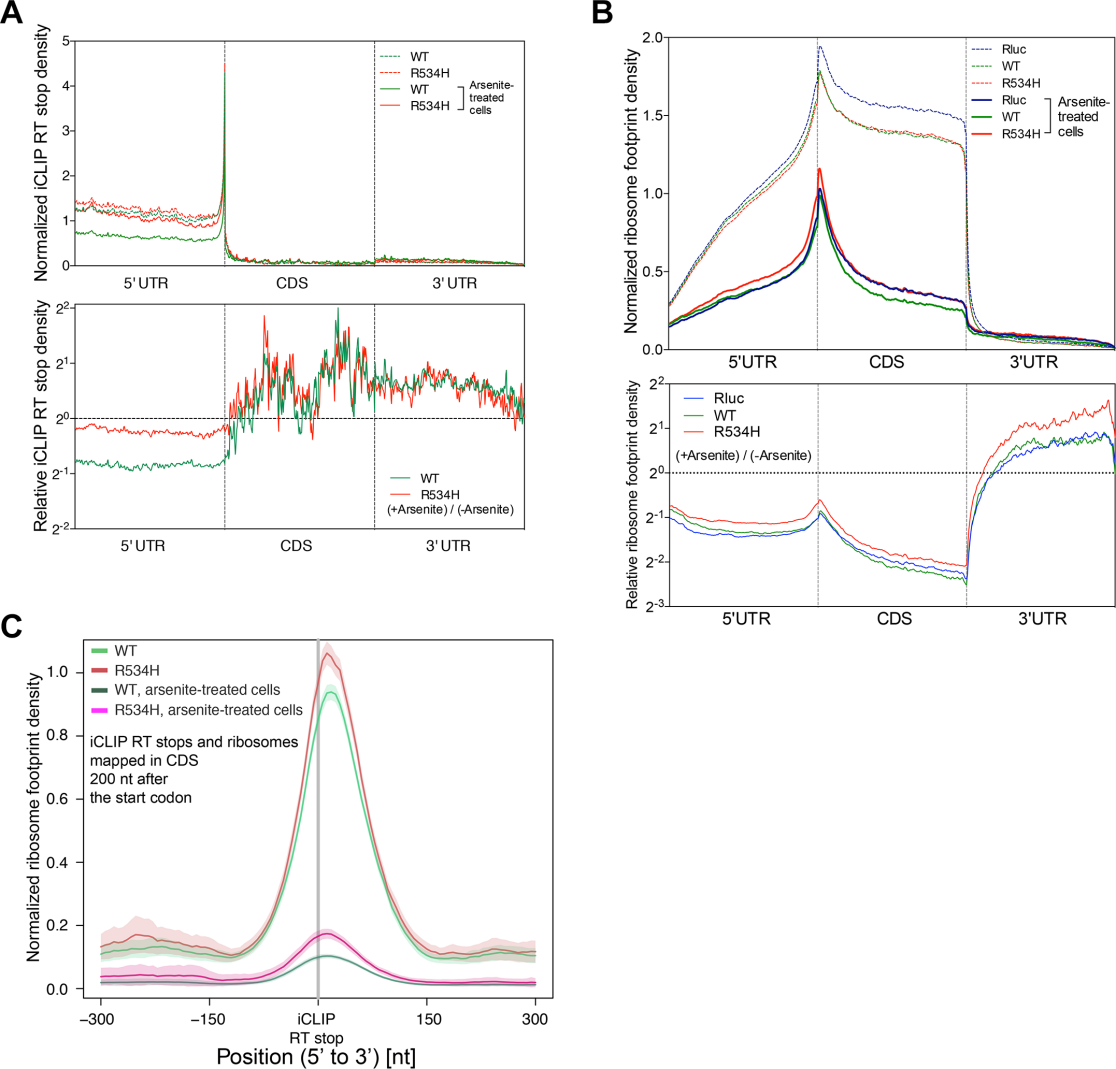
DDX3 catalytic activity is required for proper translation response to stress (**A**) (Top) Metagene analysis of normalized iCLIP RT stop density from DDX3 (green) and DDX3^R534H^ (red) iCLIP experiments with (solid lines) or without (dotted lines) arsenite treatment. 5′UTR, CDS, and 3′UTR regions were each scaled to 200 bins. iCLIP RT stops were normalized for library size and calculated as counts in 10 million and the average RT stop density is shown (Bottom) Metagene plot of iCLIP RT stop density of arsenite-treated cells relative to untreated cells from DDX3 (green) and DDX3^R534H^ (red) iCLIP experiments. (**B**) (Top) Normalized ribosome footprint density along a metagene, averaged from entire translating mRNAs, scaled to 200 bins each for 5′UTR, CDS, and 3′UTR, normalized for library size, and calculated as counts in million mapped reads. Solid lines, arsenite-treated cells. Dotted lines, arsenite-untreated cells. (Bottom) Ribosome footprint density plot showing ribosome engagement along mRNA in arsenite-treated cells expressing Rluc (blue), DDX3 (green), DDX3^R534H^ (red) relative to untreated cells. (**C**) Average ribosome footprint density (normalized for library size and shown as read count per million mapped reads) around iCLIP RT stops in CDS excluding 200 nt after the start codon. Shaded area = mean ± standard error.

Arsenite is known to repress translation through inhibiting the function of the key translation initiation factor EIF2α [22], which ultimately leads to SG formation [13, 23]. Inhibition of EIF2α function is frequently found during oncogene-induced stress as well as nutrient deprivation and other forms of metabolic stresses [24–26]. To more precisely define the DDX3 binding landscape under stress conditions, we repeated FAST-iCLIP [17] of DDX3 in arsenite-treated cells. We observed a substantial decrease of 5′ UTR binding by DDX3 and increased binding in the coding region upon exposure to arsenite (Figure 3A). However, DDX3^R534H^ attenuated the decrease in 5′UTR binding in response to arsenite while, similarly to DDX3, shifting its binding into the coding region (Figure 3A), suggesting catalytic activity of DDX3 influences its redistribution on mRNAs under stressed conditions.

Given arsenite-induced SGs contain stalled translation initiation complexes, we next correlated DDX3-mRNA interactions with ribosome density along mRNAs [27] in DDX3- and DDX3^R534H^-expressing cells with and without arsenite. We observed a substantial decrease in ribosome density along the 5′UTR and CDS after arsenite treatment, as was recently reported [24], and overexpression of DDX3 resulted in an additional modest decrease in ribosome occupancy along the CDS relative to Rluc-expressing control cells, at both baseline and in arsenite-treated conditions (Figure 3B). The overall reduction of ribosome engagement across the CDS in DDX3^R534H^-expressing cells was less pronounced than those of DDX3- and Rluc-expressing cells (Figure 3B), providing further evidence that loss of DDX3 catalytic activity confers resistance to arsenite-induced stress, perhaps through the inefficient redistribution and/or removal of ribosomes on/from mRNAs.

As DDX3 directly binds to the 18S ribosomal RNA (Figure 1E–1G), we correlated the register of DDX3 binding sites with ribosome density. Both DDX3 and DDX3^R534H^ were consistently positioned 5′ of ribosome-dense regions in the CDS (Figure 3C and Supplementary Figure S5A). This result was not due to juxtaposition of DDX3 iCLIP and ribosome footprints around the translation initiation site (TIS) (Figure 1D), as the first 200 nts downstream of the start codon were excluded in the analysis in Figure 3C and results are similar for the entire mRNA length as well as regions 200 nts upstream and downstream of the start codon (Supplementary Figure S5B–S5C). Nevertheless, DDX3 binding was dissociated from ribosome dense regions upon treatment with arsenite, and this dissociation was less apparent in DDX3^R534H^-expressing cells (Figure 3C and Supplementary Figure S5), again suggesting catalytically-impaired DDX3 dampens the response to stress. This was further supported by altered protein-protein interactions of DDX3^R534H^ in response to arsenite compared to DDX3 as measured by IP-LC/MS-MS. Proteins that co-immunoprecipitated with DDX3 after arsenite exposure showed decreased enrichment for GO terms associated with the ribosome and ribonucleoprotein complex compared to untreated cells and this effect was less pronounced in cells expressing catalytically impaired DDX3^R534H^ (Supplementary Figure S6 and Supplementary Table S3). Altogether, our data supports the importance of DDX3 catalytic activity in mounting a proper translation response to stress.

### DDX3^R534H^-expressing cells maintain translation of chromatin associated genes under stress

**Figure 4:**
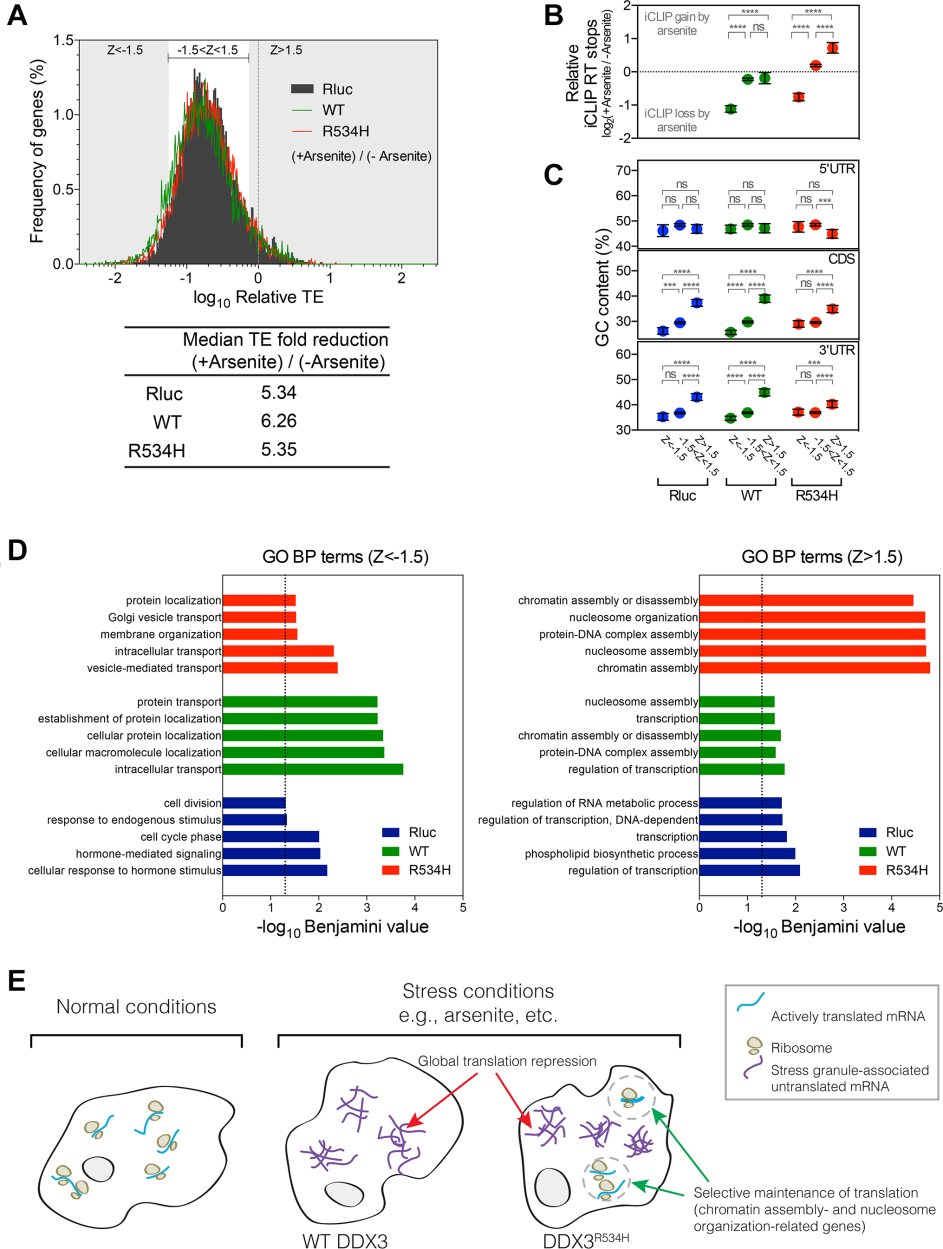
Gene-specific translation response to stress (**A**) Frequency distribution of translation efficiency (TE) of genes from cells expressing Rluc (black), DDX3 (green) or DDX3^R534H^ (red) with sodium arsenite treatment relative to no treatment. Median TE fold reduction of Rluc, DDX3, or DDX3^R534H^ –expressing cells are shown. Z score was calculated from TE of Rluc-expressing cells: grey area: Z-score > 1.5 or < -1.5 and bracketed white area: Z-score between -1.5 and 1.5. (**B**) iCLIP RT stops in arsenite-treated cells relative to those in cells without arsenite treatment. Genes were grouped by Z-scores as calculated in a. (**C**) GC content (%) in genes grouped by Z-score in a. Genes were grouped by Z-scores as calculated in a. Dashed line at zero equals to no change in iCLIP RT stops induced by arsenite. Areas above and below the zero dashed line represent iCLIP gain and loss induced by arsenite, respectively. Each gene group shows similar range of variance; Standard deviations for gene groups in DDX3-expressing cells are 1.396 (Z < −1.5), 1.495 (−1.5 < z < 1.5), and 1.891 (z > 1.5). Standard deviations for gene groups in DDX3^R534H^-expressing cells are 1.229 (Z < −1.5), 1.408 (−1.5 < z < 1.5), and 1.756 (z > 1.5). Error bars equal to mean ± 95% CI. ****P < 0.0001, ns (not significant) *P* > 0.05 calculated by ANOVA. (**D**) GO BP terms enriched in Z < −1.5 (left) and Z > 1.5 (right) gene sets as grouped in a. Dashed line, Benjamini-corrected *P*-value = 0.05. (**E**) A model for the consequence of loss of DDX3 catalytic activity in translation response. Translation is globally repressed concomitant with stress granule formation in response to stress such as arsenite, which is facilitated by wild-type DDX3 expression (middle). When the catalytically impaired DDX3^R534H^ is expressed, select transcripts escape the global translational repression caused by stress (right).

To explore whether expression of DDX3 or DDX3^R534H^ affected translation in a gene-specific manner, we calculated translation efficiency (TE) by normalizing the ribosome protected fragments to RNA-seq abundance per gene [27]. Consistent with ribosome depletion on mRNAs in response to arsenite (Figure 3B), TE was decreased substantially in control Rluc-expressing cells in response to arsenite treatment (median 5.34-fold reduction), which was further decreased by expression of wild-type DDX3 (median 6.26-fold reduction; Figure 4A). Arsenite treatment of DDX3^R534H^-expressing cells resulted in translation repression at a level comparable to that of Rluc expression (Figure 4A), further supporting that DDX3 catalytic activity facilitates translation repression in response to stress.

Despite the global decrease in TE observed after arsenite exposure, a fraction of genes escaped arsenite-induced translation repression (Figure 4A). In examining the register of genes most resistant (Z > 1.5) and sensitive (Z < −1.5) to arsenite-induced translation repression, we found the most arsenite-sensitive, translationally-repressed genes (Z < −1.5) had significantly lower levels of DDX3 and DDX3^R534H^ binding after arsenite treatment, while the bulk of mRNAs (−1.5 < Z < 1.5) had similarly bound by DDX3 after treatment with arsenite (Figure 4B). In addition, genes with higher GC content in the CDS and 3′UTR were more resistant to arsenite-mediated translation repression whereas GC content in the 5′UTR was largely not predictive (Figure 4C). These trends of the extent of translation repression relative to GC content were more apparent in DDX3-expressing cells compared to cells expressing DDX3^R534H^, again suggesting the importance of DDX3 catalytic activity in stress-induced mRNA selection and translation repression (Figure 4C). Surprisingly, arsenite-insensitive mRNAs (Z > 1.5) showed a substantial increase in binding by DDX3^R534H^, compared to DDX3 (Figure 4B), and GO terms analysis revealed these genes were enriched for “chromatin assembly” and “nucleosome organization”, suggesting a potential mechanism by which loss of DDX3 catalytic activity maintains fidelity of chromatin under stress conditions in cancer (Figure 4D and Supplementary Table S5). Notably, chromatin assembly-related gene sets were also enriched in transcriptional signatures in *DDX3X*-mutated MB patients (Supplementary Figure S7 and Supplementary Table S3) providing further evidence that translation of chromatin assembly-related genes is selectively maintained in cells expressing catalytically-impaired DDX3, perhaps as an adaptive strategy for cells sustaining oncogenic transformation and stress.

Not surprisingly, GO terms enriched in the most arsenite-sensitive, translationally repressed (Z < −1.5) mRNAs in DDX3-expressing cells were related to “protein transport” and “cellular protein localization”. These terms were less significantly enriched in DDX3^R534H^-expressing cells, perhaps providing additional selective advantage by blunting the translation repression of these important cellular functions under stress (Figure 4D and Supplementary Table S5).

### Summary

In conclusion, we have unveiled DDX3′s extensive interactions with the translational machinery at multiple levels, not only in its preference for 5′UTRs of nearly all coding RNAs but with the 18S rRNA itself. Furthermore, impairment of translation initiation is evident in human MBs harboring mutation in *DDX3X*. While the exact role of *DDX3X* mutation in establishment or maintenance of an oncogenic state is the subject of ongoing research, deregulated translation is recognized as a major driver in cancer [28] and recent studies in *MYC*-amplified medulloblastoma and glioblastoma multiforme have shown that inhibition of the translation elongation factor eEF2 allows cells to adapt to nutrient-deprived stress conditions commonly encountered in cancer [29]. Our study supports the concept that medulloblastomas have developed a key stress-adaptation strategy through alteration of a critical translation regulators, DDX3, which paradoxically tempers translation repression under stress while selectively preserving translation of key genes such as those involved with chromatin/nucleosome assembly (Figure 4E). As novel compounds targeting DDX3 have recently been discovered [30, 31] and proposed as anti-cancer agents [32], it is now imperative to understand better how cancers leverage the translational machinery to their advantage and how to best approach these novel targets therapeutically.

## MATERIALS AND METHODS

### Ethics statement and human tumor studies

All studies were performed under approval and oversight by IRB committees of Stanford University, Hospital for Sick Kids/University of Toronto and the German Cancer Research Center (DKFZ, Heidelberg), informed consent was obtained from all subjects. All human *DDX3X*-mutated SHH subtype MB samples/data that were available to us were utilized for analysis and to the extent possible, a comparison set of age- and sex-matched *DDX3X* wild type SHH subtype MB samples.

### Mammalian expression constructs

*DDX3X* cDNA (a gift of Dr. Robin Reed's laboratory, Harvard University) was PCR amplified to obtain either WT *DDX3X* CDS or missense mutant *DDX3X* CDSs via site-directed mutagenesis. *Renilla* luciferase (Rluc) CDS was in parallel PCR amplified using pRL-SV40 (Promega) as template. The PCR products were first cloned into p3X-FLAG-CMV14 (Sigma) using EcoRI and BamHI. The resulting C-terminally triple tandem FLAG-tagged fusion constructs were PCR amplified and sub-cloned into pcDNA5/FRT/TO (Life Technologies) using HindIII and XhoI. All constructs were verified with Sanger sequencing by Stanford PAN facility.

### Cell lines

Flp-In T-REx^™^ 293 cells (Life Technologies) were grown in DMEM (high glucose) supplemented with 10% (vol/vol) Tet-system approved FBS (Clontech), 2 mM L-glutamine, 1% Pen-Strep, 15 μg/ml Blasticidin S, and 100 μg/ml Zeocin at humidified 37°C and 5% CO_2_. To establish cell lines, cells were plated in a 6-well plate at the density of 0.5 × 10^6^ cells per well. On the following day, cells were transfected per well with 900 ng pOG44 (Flp recombinase plasmid, Life Technologies) and 100 ng C-terminally triple tandem FLAG-tagged DDX3X or Rluc constructs in the pcDNA5/FRT/TO backbone using Lipofectamine LTX (Life Technologies). 48 hrs post transfection, cells in each well were re-plated onto a 10- cm plate. Cells were then selected in media containing 75 μg/ml Hygromycin B and 15 μg/ml Blasticidin S but omitting Zeocin to select cells that have genomic transgene insertion at the FRT site via Flp-mediated recombination by changing media every 3 day. After 2 weeks of selection, cells were pooled and tested for their sensitivity to Zeocin. Inducibility of the FLAG-tagged construct expression by doxycycline was also tested for the Zeocin-sensitive cells with anti-FLAG Western blotting. Cells that passed both tests were used for subsequent experiments. Cell lines used in this study were not authenticated but were tested routinely for mycoplasma contamination.

### FAST-iCLIP

Flp-In T-REx^™^ 293 cells stably integrated with a doxycycline-inducible 3XFLAG-DDX3, 3XFLAG-DDX3^R534H^, or 3XFLAG-Rluc construct were induced for 8 hours with 1 μg/ml doxycycline. After induction RNA-protein interactions were stabilized with 0.3 J/cm^2^, cells collected and frozen before processing for iCLIP. The iCLIP method was performed largely as described previously [33] with the following changes. To isolate DDX3-RNA complexes 1 mg of whole cell protein lysate was incubated with 50 uL of anti-FLAG M2-conjugated agarose slurry (Sigma) for 3 hours at 4°C on rotation. Samples were washed sequentially in 1 ml for 5 min each at 4°C: twice with high stringency buffer (15 mM Tris-HCl pH 7.5, 5 mM EDTA, 2.5 mM EGTA, 1% TritonX-100, 1% Na-deoxycholate, 120 mM NaCl, 25 mM KCl), once with high salt buffer (15 mM Tris-HCl pH 7.5, 5 mM EDTA, 2.5 mM EGTA, 1% TritonX-100, 1% Na-deoxycholate, 1 M NaCl), and once with NT2 buffer (50 mM Tris-HCl pH 7.5, 150 mM NaCl, 1 mM MgCl_2_, 0.05% NP-40). The immunoprecitates were then eluted off anti-FLAG agarose beads by re-suspending each sample in 500 μL of 3XFLAG elution buffer (50 mM Tris-HCl pH 7.5, 250 mM NaCl, 0.5% NP-40, 0.1% Na-deoxycholate, 0.3 mg/ml 3XFLAG peptide (Sigma)) and rotated at 4°C for 30 minutes. The FLAG elution was repeated once more for a total of 1 ml elution. 3XFLAG-DDX3 was then captured using anti-DDX3 rabbit pAb (Abcam, ab37160) bound to Protein A Dynabeads (Life Technologies) for 3 hours at 4°C on rotation. Samples were then washed sequentially twice in 1 ml of 1% Tween-20-supplemented NT buffer and once in 1 ml of NT2 buffer. Purified DDX3-RNA complexes were processed as previously described [17, 19]. iCLIP libraries were sequenced on the Illumina HiSeq2500 platform.

### Medulloblastoma patient RNA-seq library construction and sequencing

Two micrograms of total RNA samples were arrayed into a 96-well plate and polyadenylated (PolyA+) messenger RNA (mRNA) was purified using the 96-well MultiMACS mRNA isolation kit on the MultiMACS 96 separator (Miltenyi Biotec, Germany) with on-column DNaseI-treatment as per the manufacturer's instructions. The eluted polyA + mRNA was ethanol precipitated and resuspended in 10 μL of DEPC treated water with 1:20 SuperaseIN (Life Technologies, USA). First-strand cDNA was synthesized from the purified polyA+ mRNA using the Superscript cDNA Synthesis kit (Life Technologies, USA) and random hexamer primers at a concentration of 5 μM along with a final concentration of 1 μg/μL Actinomycin D, followed by Ampure XP SPRI beads on a Biomek FX robot (Beckman-Coulter, USA). The second strand cDNA was synthesized following the Superscript cDNA Synthesis protocol by replacing the dTTP with dUTP in dNTP mix, allowing the second strand to be digested using UNG (Uracil-N-Glycosylase, Life Technologies, USA) in the post-adapter ligation reaction and thus achieving strand specificity. The cDNA was quantified in a 96-well format using PicoGreen (Life Technologies, USA) and VICTOR3V Spectrophotometer (PerkinElmer, Inc. USA). The quality was checked on a random sampling using the High Sensitivity DNA chip Assay (Agilent). The cDNA was fragmented by Covaris E210 (Covaris, USA) sonication for 55 seconds, using a Duty cycle of 20% and Intensity of 5. Plate-based libraries were prepared following the BC Cancer Agency's Michael Smith Genome Sciences Centre (BCGSC) paired-end (PE) protocol on a Biomek FX robot (Beckman-Coulter, USA). Briefly, the cDNA was purified in 96-well format using Ampure XP SPRI beads, and was subject to end-repair and phosphorylation by T4 DNA polymerase, Klenow DNA Polymerase, and T4 polynucleotide kinase respectively in a single reaction, followed by cleanup using Ampure XP SPRI beads and 3′ A-tailing by Klenow fragment (3′ to 5′ exo minus). After cleanup using Ampure XP SPRI beads, picogreen quantification was performed to determine the amount of Illumina PE adapters used in the next step of adapter ligation reaction. The adapter-ligated products were purified using Ampure XP SPRI beads, then PCR-amplified with Phusion DNA Polymerase (Thermo Fisher Scientific Inc. USA) using Illumina's PE primer set, with cycle conditions of 98°C 30 sec followed by 10–15 cycles of 98°C 10 sec, 65°C 30 sec and 72°C 30 sec, and then 72°C 5 min. The PCR products were purified using Ampure XP SPRI beads, and checked with a Caliper LabChip GX for DNA samples using the High Sensitivity Assay (PerkinElmer, Inc. USA). PCR products with a desired size range were purified using a 96-channel size selection robot developed at the BCGSC, and the DNA quality was assessed and quantified using an Agilent DNA 1000 series II assay and Quant-iT dsDNA HS Assay Kit using Qubit fluorometer (Invitrogen), then diluted to 8 nM. The final concentration was verified by Quant-iT dsDNA HS Assay. The libraries, 2 per 100 PE lane, were sequenced on the Illumina HiSeq 2000/2500 platform using v3 chemistry and HiSeq Control Software version 2.0.10.

### Alignment and SNV analysis of strand-specific RNA-seq data

Illumina paired-end RNA sequencing data was aligned to GRCh37-lite genome-plus-junctions reference [34] using BWA (version 0.5.7) [35]. This reference is a combination of GRCh37-lite assembly and exon-exon junction sequences with coordinates defined based on transcripts in Ensembl (v61), Refseq and known genes from the UCSC genome browser (both were downloaded from UCSC in November 2011; The GRCh37-lite assembly is available at http://www.bcgsc.ca/downloads/genomes/9606/hg19/1000genomes/bwa_ind/genome). BWA “aln” and “sampe” were run with default parameters, except for the inclusion of the (−s) option to disable the Smith-Waterman alignment, which is unsuitable for insert size distribution in paired-end RNA-Seq data. Finally, reads failing the Illumina chastity filter are flagged with a custom script, and duplicated reads were flagged with Picard Tools (version 1.31). After the alignment, the junction-aligned reads that mapped to exon-exon junctions were repositioned to the genome as large-gapped alignments and tagged with “ZJ:Z” by JAGuaR (version 2.0). After repositioning, hg19-aligned BAM files were split into positive-fragment and negative-fragment BAM files based on the orientation of the paired-end reads. Unmapped and improperly paired aligned reads were put into the mix-fragment BAM. SNVs were then detected on positive- and negative-split BAMs separately using SNVMix2 [36] with parameters Mb and Q30. The SNVs were further filtered to exclude those called based on 1) reference base N; 2) only 1 read supports the variant; 3) probability of heterozygous and homozygous of variant allele smaller than 0.99; 4) a position overlapping with insertions or deletions; 5) read supports from positions no more than 5 bases from read ends; 6) supports from reads only spanning an exon-exon junction; 7) more than 0.5 proportion of supporting reads were improper paired; 8) fewer than 2 proper-paired supporting reads. SNVs located in exons equal or smaller than the read length, 100 bp in this case, are a special case, because all their coverage may come from exon-exon junction spanning reads, so we also identified small-exonic SNVs that were only supported by reads that spanning exon-exon junction but passed all other 7 filtering criteria mentioned above. These SNVs were finally annotated with SnpEff [37] (Ensembl 66) and SnpSift [38] (dbSNP137 and COSMIC64).

Illumina paired-end RNA-seq data from HEK293 cells, used in ribosome profiling analysis, were preprocessed with NGSUtils [39] and reads that mapped to an rRNA library were removed. Remaining reads were mapped to the hg19 transcriptome using the STAR aligner [40]. Reads that uniquely mapped to the genome were then counted using NGSUtils and an FPKM value calculated.

For clustering and GSEA analysis, the FPKM datasets were trimmed to only the protein coding genes, followed by quantile normalization and log2 transformation of each dataset. Unsupervised clustering analysis was performed using the consensus clustering default parameters in GENE-E (http://www.broadinstitute.org/cancer/software/GENE-E/). GSEA was performed as previously described [20] using the desktop module (http://www.broadinstitute.org/gsea/index.jsp) and MsigDB gene set collections CP (canonical pathways), GO (gene ontology) and CGP (chemical and genetic perturbations).

### Western blot analysis

1 μg/ml doxycycline was added to the culture of Flp-In T-REx^™^ 293 cells stably integrated with a doxycycline-inducible 3XFLAG-DDX3, 3XFLAG-DDX3^R534H^, or 3XFLAG-Rluc construct, and cells were subsequently incubated for varying times. Primary antibodies used in this study are as follows: anti-DDX3 mouse monoclonal antibody (mAb) (a gift of Dr. Venu Raman's laboratory, The Johns Hopkins University), anti-DDX3 rabbit polyclonal antibody (pAb) (Abcam, ab37160), anti-FLAG M2 mouse mAb (Sigma, F3165), anti-FLAG rabbit pAb (Sigma, F7425), anti-β-Actin rabbit pAb (Abcam, ab8227) and anti-β-Tubulin rabbit pAb (Abcam, ab6046).

### Large-scale immunoprecipitation and mass spectrometry

Flp-In T-REx^™^ 293 cells stably integrated with a doxycycline-inducible 3XFLAG-DDX3, 3XFLAG-DDX3^R534H^, or 3XFLAG-Rluc construct were plated into five 15-cm plates per immunoprecipitation. 1 μg/ml doxycycline was added to the cells at ∼75% confluency for 8 hr-incubation. After washing twice with ice-cold PBS, cells were lysed in Buffer A (50 mM Tris-HCl (pH 7.5), 125 mM NaCl, 1.5 mM MgCl_2_, 25 mM NaF, 2 mM Na_3_VO_4_, 1% Triton X-100, 10% glycerol, and Complete Mini protease inhibitor cocktail (Roche)) [41]. After clearance of insoluble materials, 50 mg proteins were incubated overnight with 100 μl anti-FLAG M2-conjuated agarose beads (Sigma). Beads were washed 5 times with Buffer A and additional 5 times with Buffer B (50 mM Tris-HCl (pH 7.5), 125 mM NaCl, 1.5 mM MgCl_2_, 25 mM NaF, 2 mM Na_3_VO_4_, and Complete Mini protease inhibitor cocktail (Roche)) for 30 min each by rotation at 4°C [41]. Immunoprecipitates were eluted with 250 μl of 0.1 M Glycine (pH 2.5) and neutralized by adding 50 μl of 1 M Tris-HCl (pH 8.0). 12 μl of the neutralized eluents (4% equivalent of the total eluents) were run on Any kD^™^ SDS-PAGE gel (Bio-Rad) and stained by silver nitrate (Pierce). The remainder of the neutralized eluents was precipitated with 4X volumes of −80°C acetone on dry ice for at least 1 hour. Following centrifugation at 4°C, 12,000 g for 10 min, the supernatant was removed and the samples speed vac dried for 5 min. The protein pellet was reconstituted in 35 μl 0.02% of acid labile surfactant protease max (Promega), 50 mM ammonium bicarbonate. The samples were reduced using DTT (5 mM) at 50°C for 30 min followed by alkylation using propionamide (10 mM) for 30 min at room temperature. The sample volume was adjusted to 100 μL by the addition of 50 mM ammonium bicarbonate after which Trypsin/LysC (Promega) was added to a protein to protease ratio of 25:1, respectively. The samples digested overnight at 37°C, followed by an acid quench using 10 μl of 10% formic acid water. Peptides were stage-tip purified (NEST group) on C18 columns where the eluent was dried and stored at 4°C. The purified peptides were run through NanoAquity UPLC (Waters) at the rate of 300 nl per min with no pre-column. The analytical column was in house packed using 3 μM C18 reversed phase particles (Peeke Scientific) at 15 cm in length. LC acquisition time varied from 90 to 120 min gradients. A LTQ Orbitrap Velos mass spectrometer was set to acquire in DDA fashion, where the top 15 most intense precursor ions were selected for fragmentation in the ion trap. The .RAW data was converted to mgf format and searched using Byonic (Protein Metrics). The tolerances were 8-ppm precursor mass error and 0.25 Da fragment ion error searched in a target decoy approach, where the FDR was set to 1%.

### Immunofluorescence microscopy

Flp-In T-REx^™^ 293 cells stably integrated with a doxycycline-inducible 3XFLAG-DDX3, 3XFLAG-DDX3^R534H^, or 3XFLAG-Rluc construct were plated at the density of 0.5 × 10^6^ cells per well in 6-well plates. For immunofluorescence, cells were plated at the same density on coverslips placed in 6-well plates. On the following day, cells were incubated for 8 hrs with 1 μg/ml Doxycycline. 45 min before harvest, cells were treated with or without either 0.5 mM sodium arsenite or 4 μg/ml Cycloheximide. Cells were PBS washed, fixed in 4% paraformaldehyde, permeabilized in 0.1% Triton X-100 and incubated with blocking solution (PBS supplemented with 3% BSA and 10% normal goat serum), then with primary and secondary antibodies sequentially. Images were captured using a Leica DM5500 confocal microscope, and processed using ImageJ software. Primary antibodies used for immunfluorescence microscopy used are anti-FLAG M2 mouse mAb (Sigma) and anti-TIA-1 rabbit pAb (Santa Cruz Biotechnologies).

### Ribosome profiling

Ribosome profiling was performed with a modified protocol of Ingolia et al., (2009) [27] using ARTseq^™^ ribosome profiling kit (EpiCentre). Flp-In T-REx^™^ 293 cells stably integrated with a doxycycline-inducible 3XFLAG-DDX3, 3XFLAG-DDX3^R534H^, or 3XFLAG-Rluc construct were incubated with 1 μg/ml Doxycycline (8 hrs), with or without 0.5 mM sodium arsenite (for last 45 min), and with 0.1 mg/ml Cycloheximide (for last 10 min). 20 × 10^6^ cells per condition were subsequently washed and lysed both in the presence of 0.1 mg/ml Cycloheximide. Cleared lysate (∼1 ml) was treated with ARTseq Nuclease (1 Unit per 1 A_260_), aliquot (100 μl each), and snap frozen until use. RNA was extracted before (total RNA fraction from 100 μl lysates) and after MicroSpin S-400 column chromatography (mono-ribosome fraction from 200 μl lysates). After depleting rRNA (Ribo-Zero Kit, EpiCentre), the total RNA fraction was heat fragmented, and the mono-ribosome fraction was separated in denaturing PAGE gel to obtain ∼28–30 nt RNA enriched for ribosome protected mRNA fragments (RPFs). Both total RNA and RPFs were then ligated to a 3′ adapter after PNK 3′ end repair and reverse transcribed. The resulting cDNA was PAGE purified, circularized (CircLigage, EpiCentre), PCR amplified (12 index primers, 9 cycles), profiled in BioAnalyzer (Agillent), and subjected to sequencing on the Illumina HiSeq2500 platform. Reads were mapped to the hg19 transcriptome with the STAR aligner [19, 42]. The number of reads mapped to each gene was obtained using NGSUtils [40] with Ensembl gene annotations (v74). RPF reads which had a count less than 36 across all samples were eliminated. Translation efficiency (TE) was calculated for each gene by dividing FPKM of RPF seq reads by FPKM of total RNA seq reads [39].

### Ribosome density and GC content at DDX3 CLIP sites

Ribosome density was plotted at DDX3 iCLIP sites using NGSPLOT [43]. The coverage of ribosomal protected fragments was determined by contracting the fragments to 30 bp, using only reads with a mapping quality of 20 or greater. The coverage vectors were then normalized by total library size. The average plots were generated following the removal of 0.1% of outliers. To prevent frequently occurring DDX3 binding sites from skewing the ribosome density average clusters of sites were merged. iCLIP sites within 100 bp of another site were combined into a single region by taking the center of the nearby iCLIP sites. GC content at each of the iCLIP events was calculated using a 10 bp sliding window, with the center occupying the 6th bp. The GC content vectors of each iCLIP site were then averaged. Sequences were extracted from the hg19 genome using bedtools. The iCLIP sites were merged to prevent bias as previously described for calculating ribosome density.

### Accession codes

The patient RNA-seq provisional European Genome-phenome Archive (EGA) numbers is EGAD00001001210. iCLIP-seq, total RNA-seq, ribosome profiling datasets will be accessible through the Gene Expression Omnibus (GEO) data repository under accession number GSE70804.

## SUPPLEMENTARY MATERIALS FIGURES AND TABLES













## References

[1] Linder P, Jankowsky E (2011). From unwinding to clamping - the DEAD box RNA helicase family. Nat Rev Mol Cell Biol.

[2] Northcott PA, Jones DTW, Kool M, Robinson GW, Gilbertson RJ, Cho YJ, Pomeroy SL, Korshunov A, Lichter P, Taylor MD, Pfister SM (2012). Medulloblastomics: the end of the beginning. Nat Rev Cancer.

[3] Kool M, Jones DTW, Jäger N, Northcott PA, Pugh TJ, Hovestadt V, Piro RM, Esparza LA, Markant SL, Remke M, Milde T, Bourdeaut F, Ryzhova M (2014). Genome sequencing of SHH medulloblastoma predicts genotype-related response to Smoothened inhibition. Cancer Cell.

[4] Pugh TJ, Weeraratne SD, Archer TC, Pomeranz Krummel DA, Auclair D, Bochicchio J, Carneiro MO, Carter SL, Cibulskis K, Erlich RL, Greulich H, Lawrence MS, Lennon NJ (2012). Medulloblastoma exome sequencing uncovers subtype-specific somatic mutations. Nature.

[5] Floor SN, Condon KJ, Sharma D, Jankowsky E, Doudna JA (2016). Autoinhibitory Interdomain Interactions and Subfamily-specific Extensions Redefine the Catalytic Core of the Human DEAD-box Protein DDX3. J Biol Chem.

[6] Epling LB, Grace CR, Lowe BR, Partridge JF, Enemark EJ (2015). Cancer-associated mutants of RNA helicase DDX3X are defective in RNA-stimulated ATP hydrolysis. J Mol Biol.

[7] Soto-Rifo R, Rubilar PS, Limousin T, de Breyne S, Décimo D, Ohlmann T (2012). DEAD-box protein DDX3 associates with eIF4F to promote translation of selected mRNAs. EMBO J.

[8] Chuang RY, Weaver PL, Liu Z, Chang TH (1997). Requirement of the DEAD-Box protein ded1p for messenger RNA translation. Science.

[9] Hilliker A, Gao Z, Jankowsky E, Parker R (2011). The DEAD-box protein Ded1 modulates translation by the formation and resolution of an eIF4F-mRNA complex. Mol Cell.

[10] Abaeva IS, Marintchev A, Pisareva VP, Hellen CUT, Pestova TV (2011). Bypassing of stems versus linear base-by-base inspection of mammalian mRNAs during ribosomal scanning. EMBO J.

[11] Shih J-W, Tsai T-Y, Chao C-H, Wu Lee Y-H (2008). Candidate tumor suppressor DDX3 RNA helicase specifically represses cap-dependent translation by acting as an eIF4E inhibitory protein. Oncogene.

[12] Anderson P, Kedersha N (2009). RNA granules: post-transcriptional and epigenetic modulators of gene expression. Nat Rev Mol Cell Biol.

[13] Buchan JR, Parker R (2009). Eukaryotic stress granules: the ins and outs of translation. Mol Cell.

[14] Shih JW, Wang WT, Tsai TY, Kuo CY, Li HK, Wu Lee YH (2012). Critical roles of RNA helicase DDX3 and its interactions with eIF4E/PABP1 in stress granule assembly and stress response. Biochem J.

[15] Lai M-C, Lee Y-HW, Tarn W-Y (2008). The DEAD-box RNA helicase DDX3 associates with export messenger ribonucleoproteins as well as tip-associated protein and participates in translational control. Mol Biol Cell.

[16] Goulet I, Boisvenue S, Mokas S, Mazroui R, Côté J (2008). TDRD3, a novel Tudor domain-containing protein, localizes to cytoplasmic stress granules. Hum Mol Genet.

[17] Flynn RA, Martin L, Spitale RC, Do BT, Sagan SM, Zarnegar B, Qu K, Khavari PA, Quake SR, Sarnow P, Chang HY (2015). Dissecting noncoding and pathogen RNA-protein interactomes. RNA.

[18] Anger AM, Armache J-P, Berninghausen O, Habeck M, Subklewe M, Wilson DN, Beckmann R (2013). Structures of the human and Drosophila 80S ribosome. Nature.

[19] Calo E, Flynn RA, Martin L, Spitale RC, Chang HY, Wysocka J (2015). RNA helicase DDX21 coordinates transcription and ribosomal RNA processing. Nature.

[20] Subramanian A, Tamayo P, Mootha VK, Mukherjee S, Ebert BL, Gillette MA, Paulovich A, Pomeroy SL, Golub TR, Lander ES, Mesirov JP (2005). Gene set enrichment analysis: a knowledge-based approach for interpreting genome-wide expression profiles. Proc Natl Acad Sci USA.

[21] Soto-Rifo R, Ohlmann T (2013). The role of the DEAD-box RNA helicase DDX3 in mRNA metabolism. Wiley Interdiscip Rev RNA.

[22] McEwen E, Kedersha N, Song B, Scheuner D, Gilks N, Han A, Chen JJ, Anderson P, Kaufman RJ (2005). Heme-regulated inhibitor kinase-mediated phosphorylation of eukaryotic translation initiation factor 2 inhibits translation, induces stress granule formation, and mediates survival upon arsenite exposure. J Biol Chem.

[23] Kedersha N, Ivanov P, Anderson P (2013). Stress granules and cell signaling: more than just a passing phase?. Trends Biochem Sci.

[24] Andreev DE, O'Connor PBF, Fahey C, Kenny EM, Terenin IM, Dmitriev SE, Cormican P, Morris DW, Shatsky IN, Baranov PV (2015). Translation of 5′ leaders is pervasive in genes resistant to eIF2 repression. Elife.

[25] Sonenberg N, Hinnebusch AG (2009). Regulation of translation initiation in eukaryotes: mechanisms and biological targets. Cell.

[26] Liu B, Qian S-B (2014). Translational reprogramming in cellular stress response. Wiley Interdiscip Rev RNA.

[27] Ingolia NT, Ghaemmaghami S, Newman JRS, Weissman JS (2009). Genome-wide analysis *in vivo* of translation with nucleotide resolution using ribosome profiling. Science.

[28] Silvera D, Formenti SC, Schneider RJ (2010). Translational control in cancer. Nat Rev Cancer.

[29] Leprivier G, Remke M, Rotblat B, Dubuc A, Mateo A-RF, Kool M, Agnihotri S, El-Naggar A, Yu B, Somasekharan SP, Faubert B, Bridon G, Tognon CE (2013). The eEF2 kinase confers resistance to nutrient deprivation by blocking translation elongation. Cell.

[30] Floor SN, Barkovich KJ, Condon KJ, Shokat KM, Doudna JA (2016). Analog sensitive chemical inhibition of the DEAD-box protein DDX3. Protein Sci.

[31] Bol GM, Vesuna F, Xie M, Zeng J, Aziz K, Gandhi N, Levine A, Irving A, Korz D, Tantravedi S, Heerma van Voss MR, Gabrielson K, Bordt EA (2015). Targeting DDX3 with a small molecule inhibitor for lung cancer therapy. EMBO Mol Med.

[32] Bol GM, Xie M, Raman V (2015). DDX3, a potential target for cancer treatment. Mol Cancer. BioMed Central.

[33] Katoh K, Standley DM (2013). MAFFT multiple sequence alignment software version 7: improvements in performance and usability. Mol Biol Evol.

[34] Butterfield YS, Kreitzman M, Thiessen N, Corbett RD, Li Y, Pang J, Ma YP, Jones SJM, Birol I (2014). JAGuaR: junction alignments to genome for RNA-seq reads. PLoS One.

[35] Li H, Durbin R (2009). Fast and accurate short read alignment with Burrows-Wheeler transform. Bioinformatics.

[36] Goya R, Sun MGF, Morin RD, Leung G, Ha G, Wiegand KC, Senz J, Crisan A, Marra MA, Hirst M, Huntsman D, Murphy KP, Aparcio S (2010). SNVMix: predicting single nucleotide variants from next-generation sequencing of tumors. Bioinformatics.

[37] Cingolani P, Platts A, Wang LL, Coon M, Nguyen T, Wang L, Land SJ, Lu X, Ruden DM (2012). A program for annotating and predicting the effects of single nucleotide polymorphisms, SnpEff: SNPs in the genome of Drosophila melanogaster strain w1118;iso-2; iso-3. Fly (Austin).

[38] Cingolani P, Patel VM, Coon M, Nguyen T, Land SJ, Ruden DM, Lu X (2012). Using Drosophila melanogaster as a Model for Genotoxic Chemical Mutational Studies with a New Program, SnpSift. Front Genet.

[39] Breese MR, Liu Y (2013). NGSUtils: a software suite for analyzing and manipulating next-generation sequencing datasets. Bioinformatics.

[40] Dobin A, Davis CA, Schlesinger F, Drenkow J, Zaleski C, Jha S, Batut P, Chaisson M, Gingeras TR (2013). STAR: ultrafast universal RNA-seq aligner. Bioinformatics.

[41] Oh S, Kato M, Zhang C, Guo Y, Beachy PA (2015). A Comparison of Ci/Gli Activity as Regulated by Sufu in *Drosophila* and Mammalian Hedgehog Response. PLoS One.

[42] Martin L, Meier M, Lyons SM, Sit RV, Marzluff WF, Quake SR, Chang HY (2012). Systematic reconstruction of RNA functional motifs with high-throughput microfluidics. Nat Meth.

[43] Shen L, Shao N, Liu X, Nestler E (2014). ngs.plot: Quick mining and visualization of next-generation sequencing data by integrating genomic databases. BMC Genomics.

