# Supplementary material for: Medulloblastoma-associated DDX3 variant selectively alters the translational response to stress
Source: Oncotarget. 2016 Apr 5;7(19):28169–82. doi: 10.18632/oncotarget.8612 (PMC5053718; doi:10.18632/oncotarget.8612)
Supplement: Supplementary file 1 [file oncotarget-07-28169-s001.pdf]

Medulloblastoma-associated DDX3 variant selectively alters the translational response to stress

Supplementary Materials

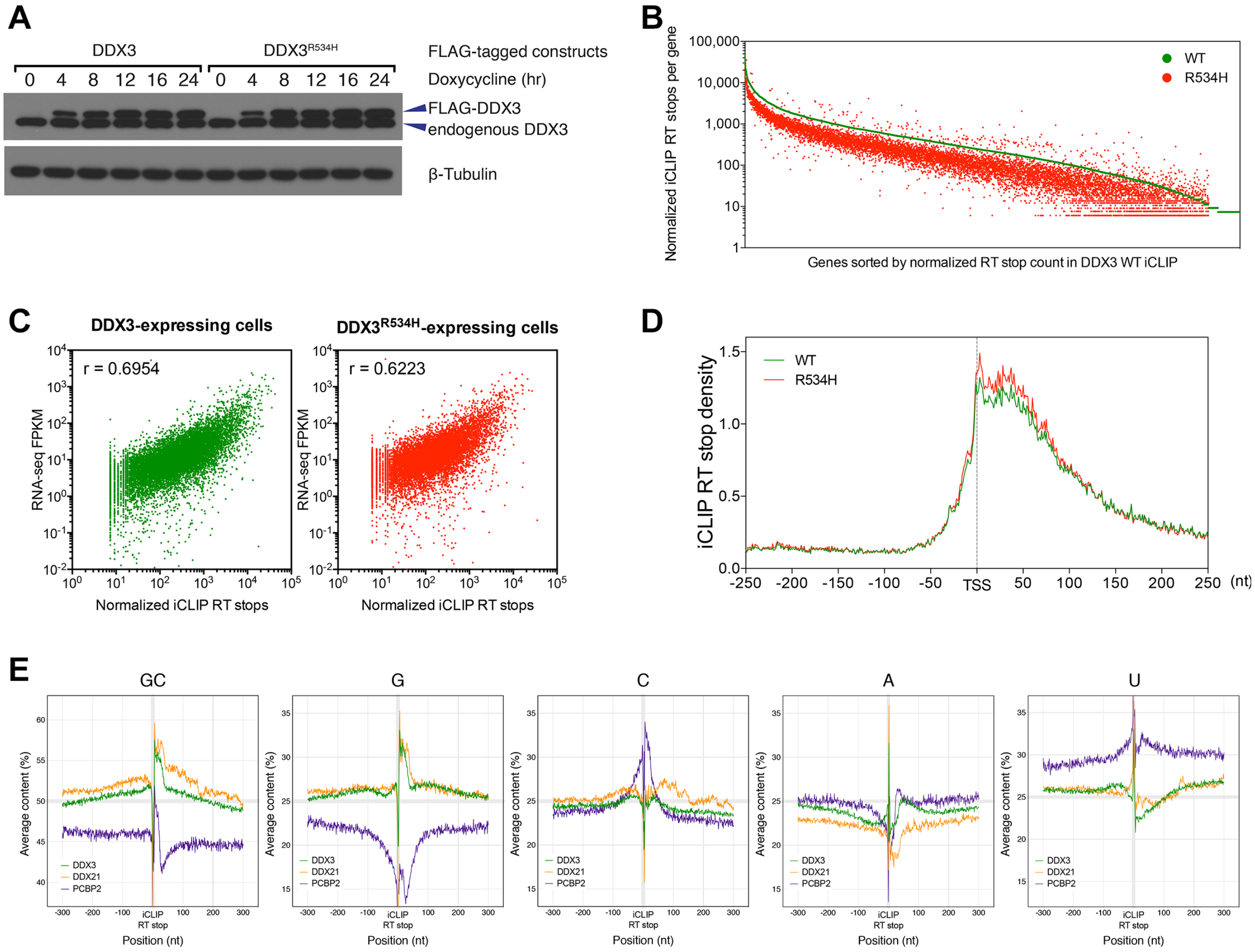

Supplementary Figure S1: DDX3 binds almost all expressed mRNAs through guanine-rich sequences. (**A**) Western blot analysis of doxycycline-mediated induction of FLAG-tagged DDX3 (WT) or DDX3^{R534H} in Flp-In T-REx 293 cells. β-Tubulin, loading control. (**B**) Distribution of normalized iCLIP RT stops per gene sorted by decreasing order of RT stops in DDX3 iCLIP. iCLIP RT stops were normalized for library size and calculated as counts in 10 million. (**C**) DDX3 (left) and DDX3^{R534H} (right) iCLIP RT stop counts per gene correlates with gene expression as measured by RNA-seq Fragments Per Kilobase of transcript per Million mapped reads (FPKM). iCLIP RT stops were normalized for library size and calculated as counts in 10 million. r, correlation coefficient. (**D**) RT stop density of DDX3 (green) and DDX3^{R534H} (red) iCLIP experiments centered at transcription start sites (TSS) showing 250 nts up and downstream. (**E**) Average content (%) of GC or individual nucleotide content around iCLIP RT stops for DDX3, DDX21 [19], and PCBP2 [19] iCLIP RT stops. G, guanidine. C, cytosine. A, adenosine. U, uridine.

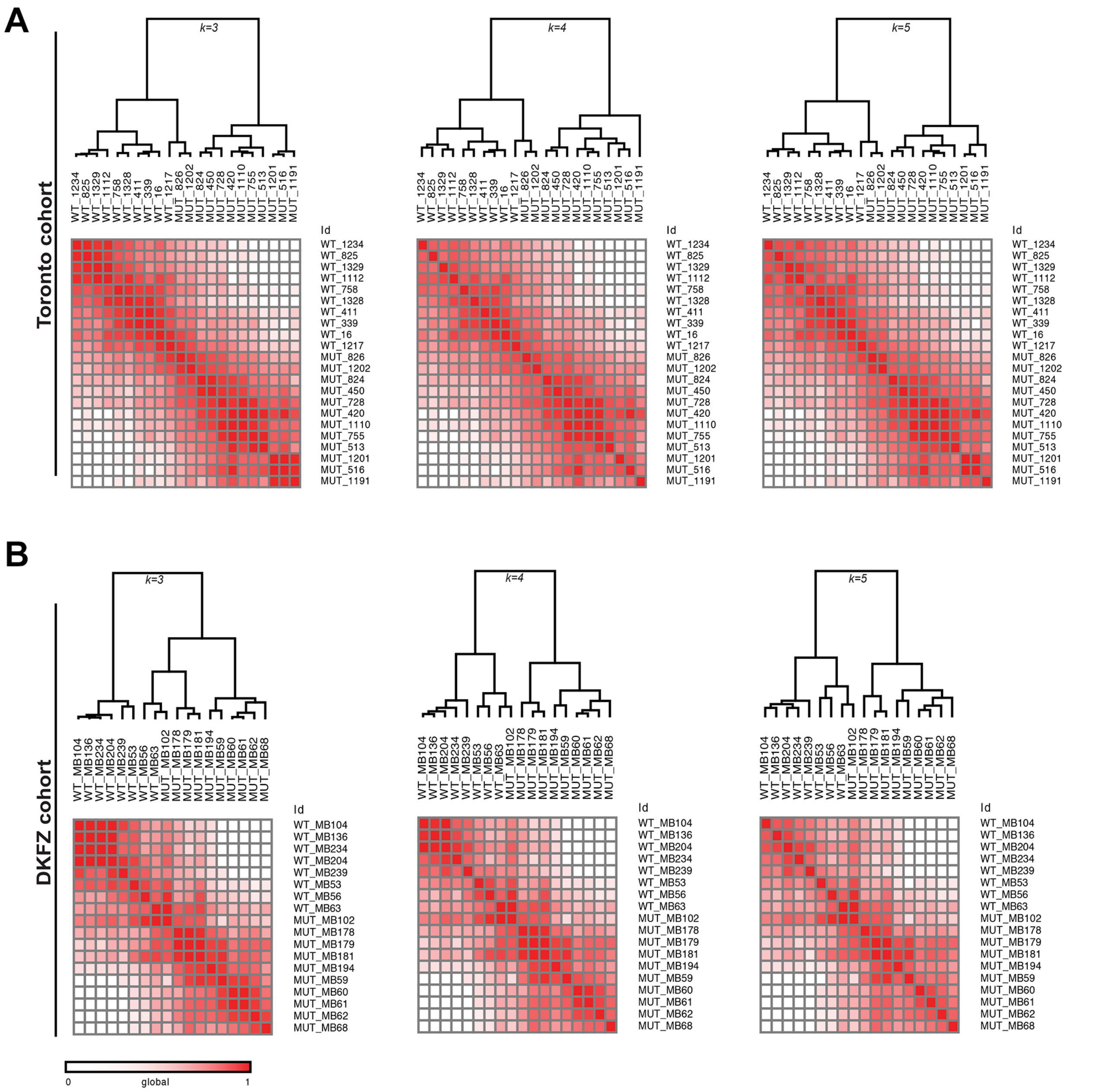

Supplementary Figure S2: Unsupervised consensus clustering results, k = 3–5, of gene expression data derived from RNA-seq data generated of two independent, non-overlapping cohorts of SHH subtype medulloblastomas. (**A**) Toronto cohort (**B**) German Cancer Research Center (DKFZ) cohort.

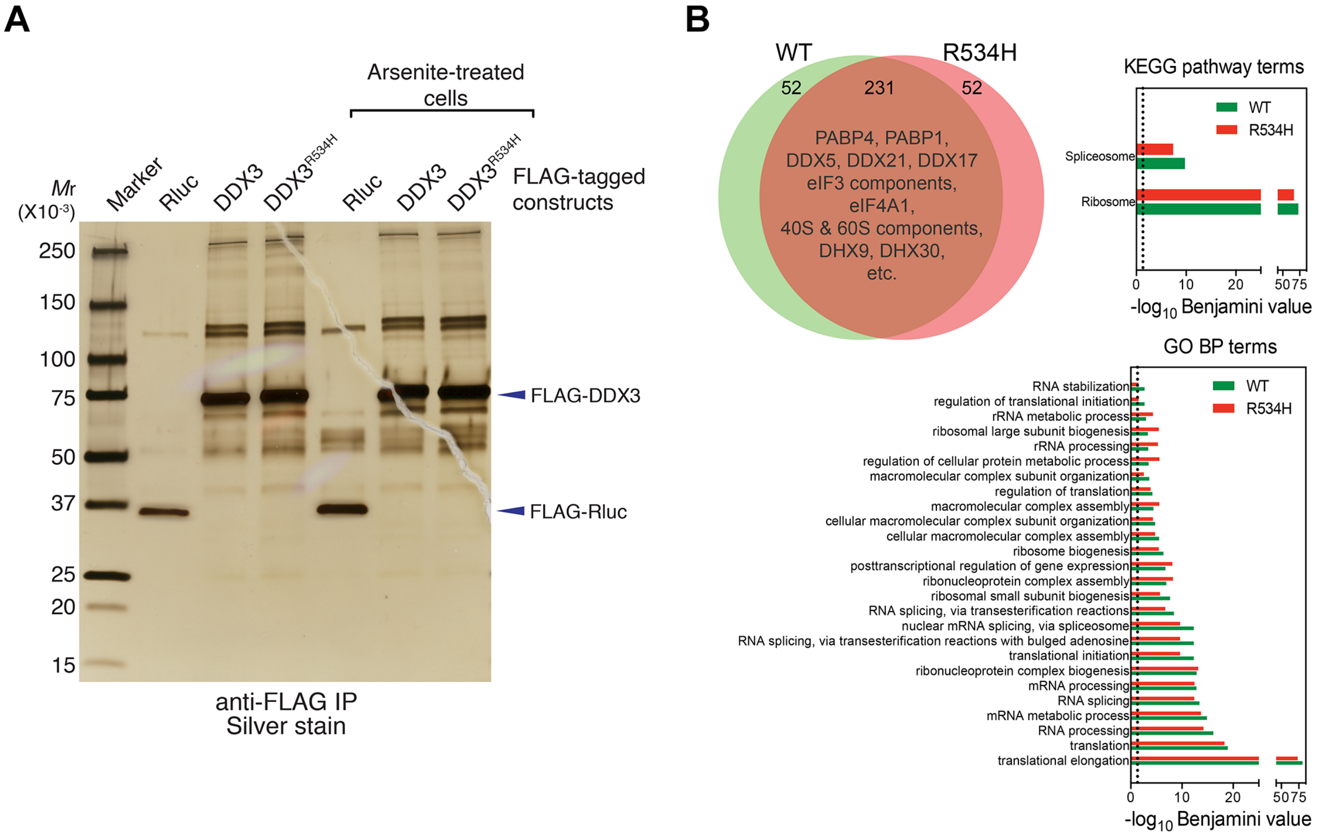

Supplementary Figure S3: DDX3 interacts with proteins involved in translation and splicing independent of its catalytic activity. (**A**) Silver stained SDS-PAGE gel resolving protein complexes that contain FLAG-tagged Renilla luciferase (Rluc), DDX3 (WT), or DDX3^{R534H}. 4% of anti-FLAG immunoprecipitates were run in the SDS-PAGE gel. The remainder of the samples was subjected to LC-MS/MS. (**B**) Specific proteins associated with DDX3 (WT) or DDX3^{R534H} but not with Rluc revealed by LC-MS/MS. (Left) Venn diagram showing number of proteins for each category. (Right) GO Biological Process (BP) and KEGG pathway terms for the proteins co-purified with DDX3 proteins are shown. Dashed line, Benjamini-corrected *P*-value = 0.05.

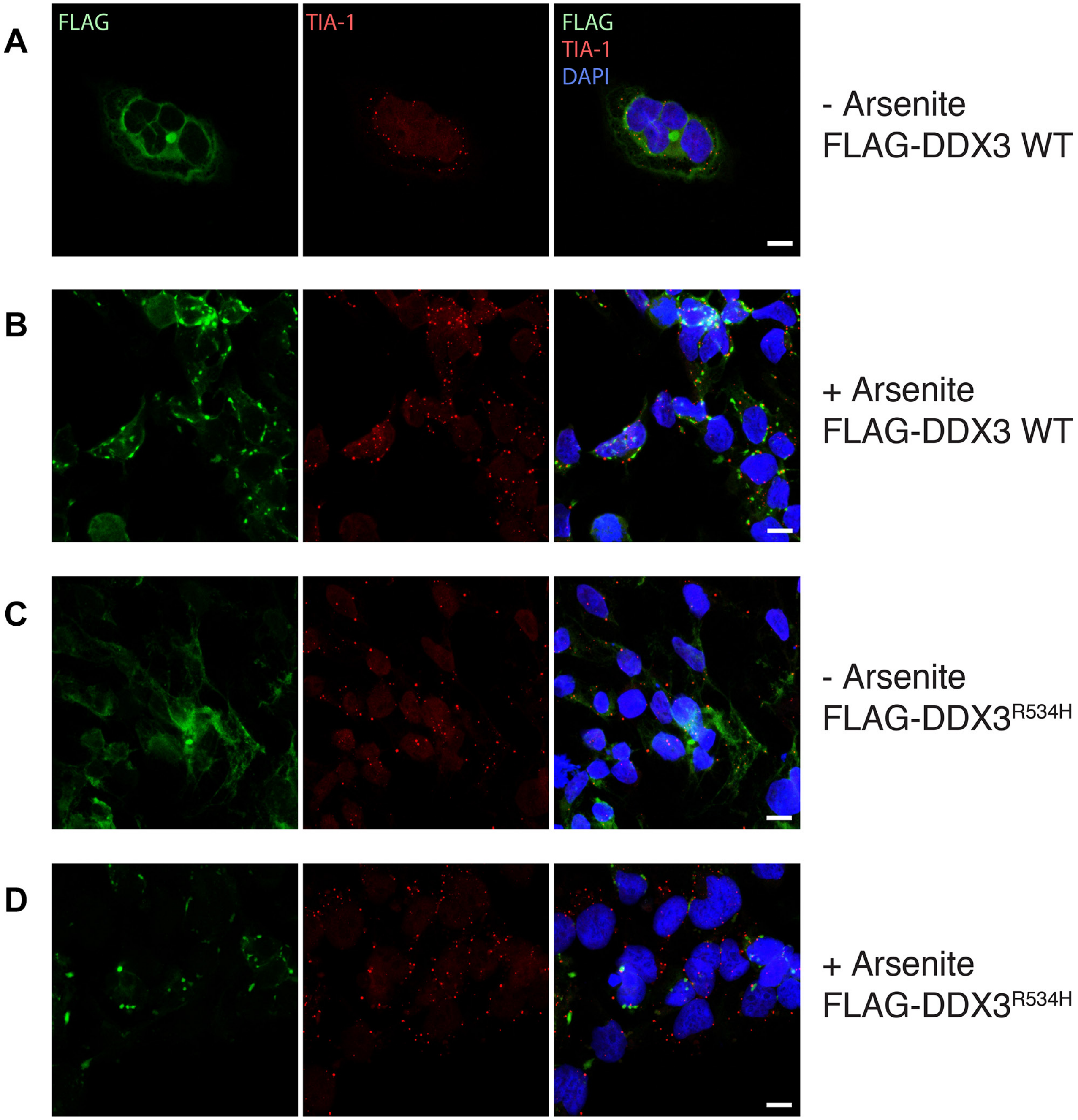

Supplementary Figure S4: Arsenite induces stress granules independently of DDX3^{R534H} expression. FLAG-DDX3 (**A** and **B**) and DDX3^{R534H} (**C** and **D**) were expressed with (b and d) or without (a and c) sodium arsenite treatment. TIA-1, a stress granule marker. Scale bar = 10 μm.

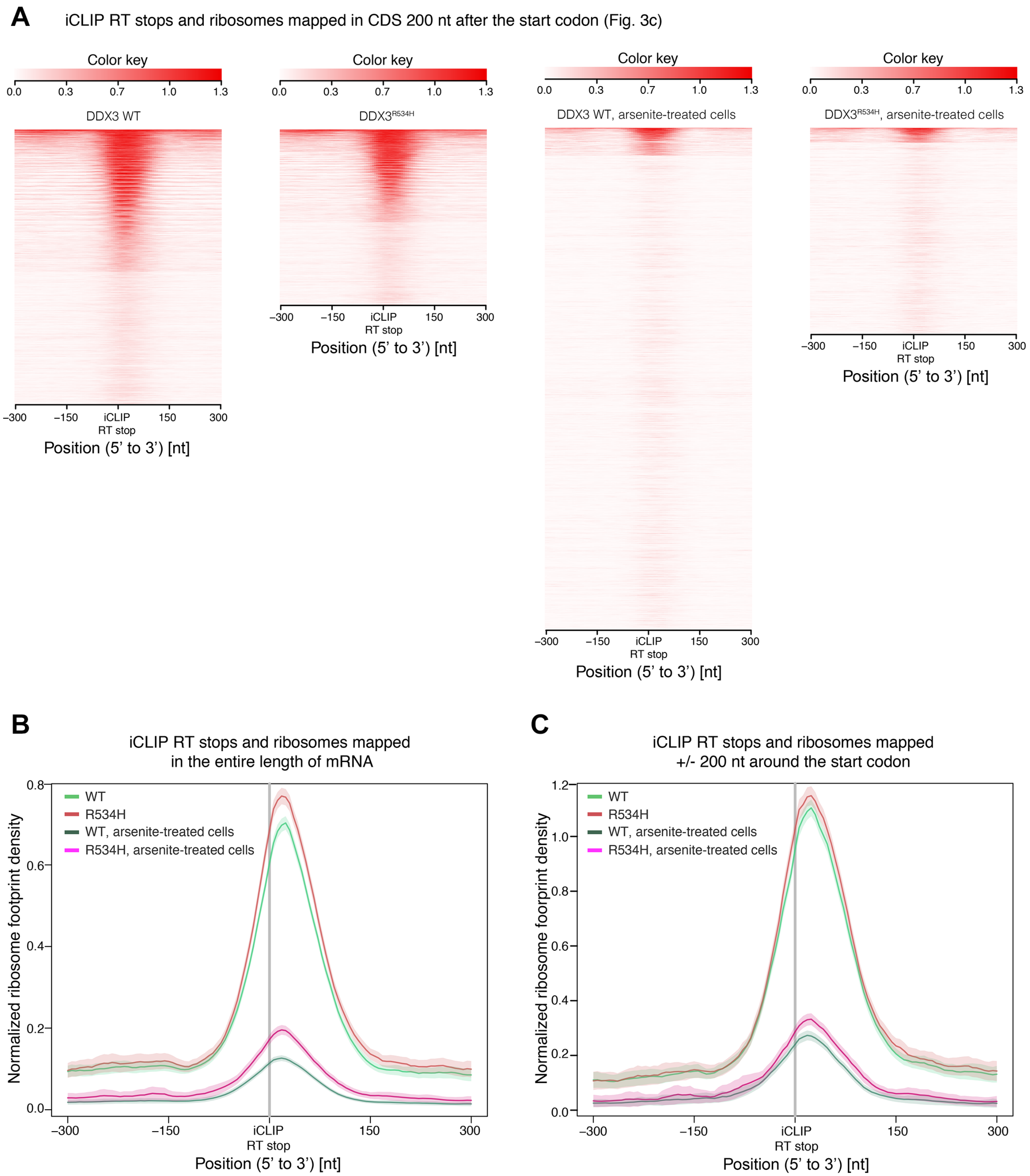

Supplementary Figure S5: DDX3 selectively binds to mRNAs upstream of 3′ flanking ribosome-dense regions. (**A**) Ribosome densitiy heat maps for genes rank order sorted around DDX3X iCLIP RT stops in CDS after start codon, which were averaged and plotted in Figure 3C. (**B** and **C**) Average ribosome footprint density (read count per million mapped reads) around iCLIP RT stops mapped in entire mRNA length (b) and in regions 200 nt upstream and downstream of start codon (c). Shaded area = mean ± standard error.

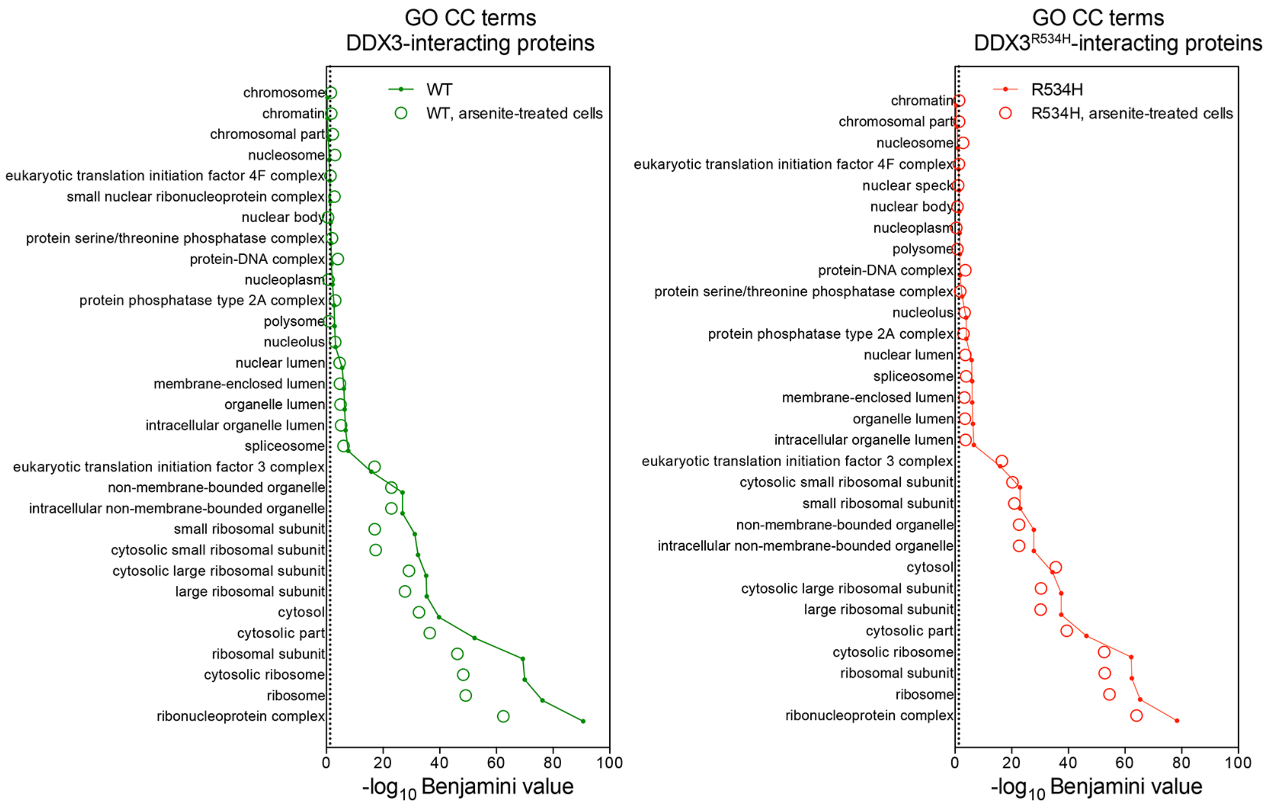

Supplementary Figure S6: Uncoupling of DDX3-ribosome interactions upon arsenite exposure are less pronounced in DDX3^{R534H}-expressing cells. GO terms (Cellular Component, CC) enriched in proteins co-purified with FLAG-DDX3 (left) and DDX3^{R534H} (right) with or without arsenite treatment. Dashed line, Benjamini-corrected *P*-value = 0.05.

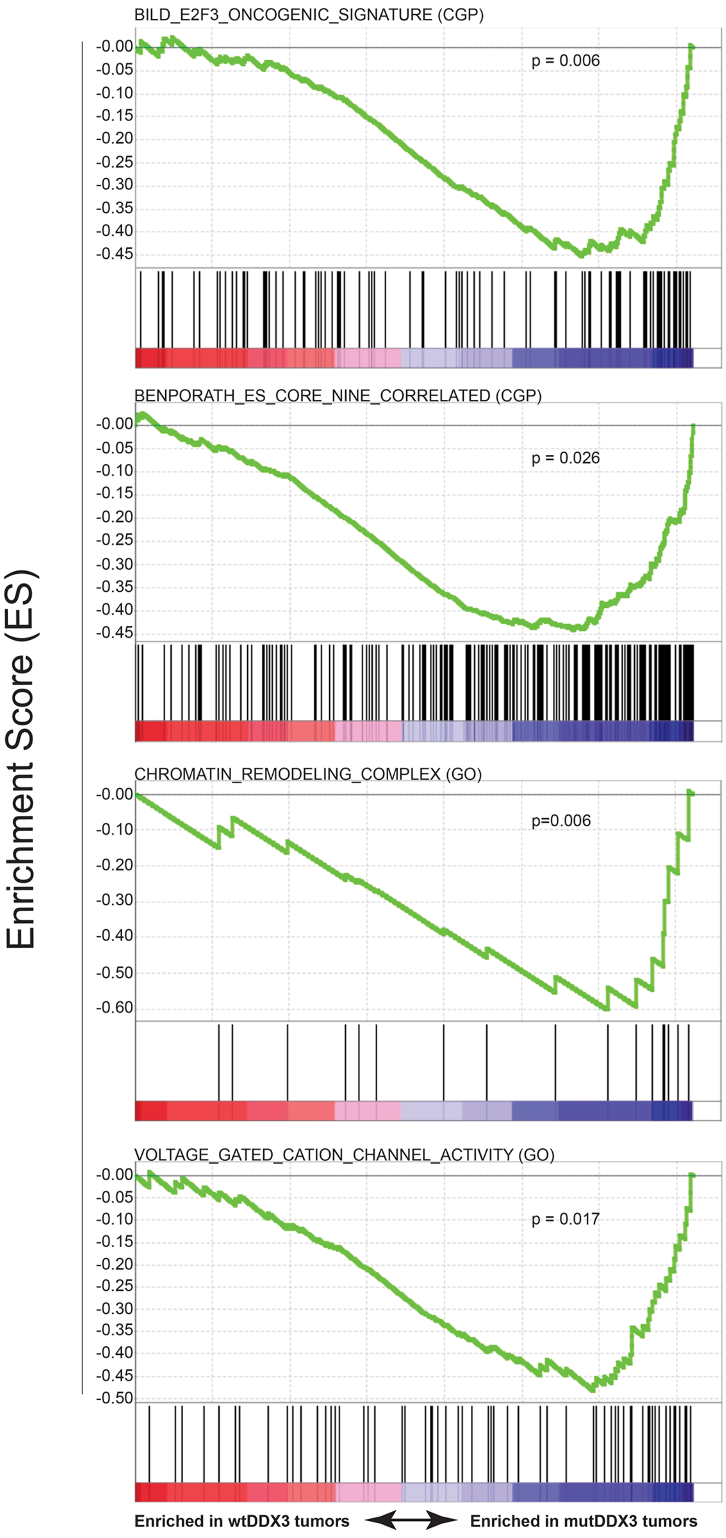

Supplementary Figure S7: Selected enrichment plots for genes enriched in *DDX3X*-mutated medulloblastoma cases relative medulloblastomas without *DDX3X* mutations.

Supplementary Table S1: List of protein-conding genes of which transcripts are bound by DDX3

Supplementary Table S2: Patient sample information for RNA-sequencing

Supplementary Table S3: Gene Set Enrichment Analysis of medulloblastomas with versus without *DDX3X* mutations

Supplementary Table S4: List of proteins co-immunopurified with DDX3

Supplementary Table S5: List of GO terms enriched in gene sets that are sensitive (Z < –1.5) and insensitive (Z > 1.5) to arsenite-mediated translation repression
